# Supplementary material for: ChIPbinner: an R package for analyzing broad histone marks binned in uniform windows from ChIP-Seq or CUT&RUN/TAG data
Source: BMC Bioinformatics. 2025 Mar 24;26:89. doi: 10.1186/s12859-025-06103-6 (PMC11934474; doi:10.1186/s12859-025-06103-6)
Supplement: Supplementary file 1 — Additional file 1: ChIPbinner source package v0.99.2 [file 12859_2025_6103_MOESM1_ESM.zip › Supplement/Additional_file_1_ChIPbinner_0.99.2.tar.gz!/ChIPbinner/inst/doc/ChIPbinner_workflow.html]

Analyzing binned ChIP-seq/Cut&Run data with ChIPbinner


# Analyzing binned ChIP-seq/Cut&Run data with ChIPbinner

```
library(ChIPbinner)
```

# Database for ChIPbinner

Only toy datasets, which have been largely downsampled, are included
with the package. For complete example datasets please see
ChIPbinner
database.

# Example workflow for generating binned BED files

For input into ChIPbinner, the sequenced samples will need to be
binned into windows, and formatted as a
BED file. One of the
tools that can be used to perform this task is
BEDtools, which
can convert an aligned sequence file, typically found in a
BAM
format, into a binned BED file as demonstrated below.

Reference windows ($WINDOW\_REF) can be found in the
ChIPbinner
database.

```
# convert aligned BAM file to BED file
bedtools bamtobed -i ${sample}.sorted.bam | sort -k1,1 -k2,2n > ${sample}.bed
# intersect sample BED file with binned reference files
bedtools intersect -a $WINDOW_REF -b ${sample}.bed -sorted -c -nonamecheck > $OUTPUT_NAME.binned.bed
# example for binning Cal27 WT sample into 10kb windows
bedtools bamtobed -i Cal27.WT.H3K36me2.sorted.bam | sort -k1,1 -k2,2n > Cal27.WT.H3K36me2.bed
bedtools intersect -a hg38.10kb.windows.bed -b Cal27.WT.H3K36me2.bed -sorted -c -nonamecheck > Cal27.WT.H3K36me2.10kb.bed
```

# Filtering bins depleted in signal across samples

Using the `filter_low_counts` function, the user can
remove bins with consistently low raw read counts (e.g., for a given
bin, if all four samples have a raw read count less than 100, it is
filtered out). This filtering step reduces computational time and
enhances the power to detect differential binding.

```
filter_low_counts(out_dir, hg38, sample_bedfiles = c("NSD1KO_rep1.bed",
    "NSD1KO_rep2.bed", "WT_rep1.bed", "WT_rep2.bed"), cutoff = 100)
```

# Normalizing raw counts by size factors

The user can choose to normalize the raw counts in their BED files
using
DESeq2’s
median ratio method or
edgeR’s
TMM. In this case, DESeq2’s median ratio method will divide the raw
bin counts by sample-specific size factors, which are calculated by
determining the median of the ratio of the raw bin counts for each bin
relative to the geometric mean per bin across all samples. Conversely,
edgeR’s TMM method will compute the TMM factor by considering one sample
as a reference sample and the other as test samples. The sample with the
count-per-million upper quartile closest to the mean upper quartile is
designated as the reference. For each test sample, TMM is calculated as
the weighted mean of log ratios between the test sample and the
reference, excluding bins with the highest coverage and those with the
largest log ratios. EdgeR assumes that there are few differential
changes, so the TMM should be close to 1. If it’s not, the TMM value
indicates the correction factor that should be applied to the library
sizes, resulting in what is referred to as the effective library size.
The normalized read counts are then calculated by dividing the raw bin
counts by the effective library size.

In addition to correcting for technical biases arising from
differences in sequencing depth across samples, both methods utilize
normalization factors to address outlier regions that could distort
comparisons of read counts per bin across samples. Thus, both methods
are robust to outliers and bins with extremely high or low coverage.
However, it is important to note that both methods assume most bins are
not differentially covered (or differentially bound) between samples.
 **While these normalization methods enhance robustness against outlier
regions, it also reduces sensitivity to detecting positive instances of
differential changes. Therefore, users should exercise caution when
applying these normalization techniques as they may reduce the power to
detect true biological changes for a given histone mark.**

Multiple samples per condition (treated and wildtype) can be provided
for this normalization. The function will then output the normalized
counts in separate BED files for each sample. The user can then input
these files into the next step — generating normalized bigWig files —
where additional normalization and/or scaling of the signal can be
performed.

```
# using DESeq2's median ratio method
apply_normFactors(norm_method = "DESeq2", out_dir = "out_dir",
    genome_assembly = "mm10", treated_sample_bedfiles = c("Cal27_NSD1KO_H3K36me2_rep1.10kb.bed",
        "Cal27_NSD1KO_H3K36me2_rep2.10kb.bed"), wildtype_sample_bedfiles = c("Cal27_WT_H3K36me2_rep1.10kb.bed",
        "Cal27_WT_H3K36me2_rep2.10kb.bed"), treated_condition_label = "NSD1KO",
    wildtype_condition_label = "WT")
# using edgeR's TMM
apply_normFactors(norm_method = "edgeR", out_dir = "out_dir",
    genome_assembly = "mm10", treated_sample_bedfiles = c("Cal27_NSD1KO_H3K36me2_rep1.10kb.bed",
        "Cal27_NSD1KO_H3K36me2_rep2.10kb.bed"), wildtype_sample_bedfiles = c("Cal27_WT_H3K36me2_rep1.10kb.bed",
        "Cal27_WT_H3K36me2_rep2.10kb.bed"), treated_condition_label = "NSD1KO",
    wildtype_condition_label = "WT")
```

# Generating normalized bigwig files

When you bin your files into windows, especially from aligned (BAM or
SAM) files, they are typically raw read counts. They will need to be
normalized by the input (highly recommended for ChIP-seq data) and
likely scaled by some factor (we recommend using a normalization scaling
factor like genome-wide modification percentage information obtained
from mass spectrometry, Drosophila spike-in or any other types of
spike-in). This step will have to be performed prior to moving on to the
rest of the workflow.

Here, we are generating normalized bigwig files for 10kb-binned files
(in BED format) for a head and neck squamous cell carcinoma (HNSCC) line
(Cal27) for the broad histone mark H3K36me2 generated via ChIP-seq. The
lysine 36 methyl transferase NSD1 was knocked out in the sample
(NSD1-KO) and is being compared to the wildtype (WT) cell line. Please
see
Farhangdoost
et al. 2021 for more details.

**Important note 1:** For ChIPbinner, only two genome assemblies
can be used: `hg38` or `mm10`. We will be using
the `hg38` assembly for this analysis.

**Important note 2:** If you’ve previously ran
`apply_normFactors` on the BED files, then please use the
parameter `depth_norm = FALSE`  to avoid redundantly
normalizing the read counts by library size.

If you’ve ran the code chunk successfully, your resulting bigwig
files will be found in the `output_directory` that you
specified when running the function. The
`immunoprecipitated_binned_file` refers to the
immunoprecipitated sample for ChIP-seq or the targeted, enriched sample
for CUT&RUN/TAG. The `input_binned_file` refers to the
genomic input for ChIP-seq samples or the IgG control sample for
CUT&RUN/TAG.

```
# generate normalized bigwig for WT sample
norm_bw(out_dir = "output_directory", genome_assembly = "hg38",
    immunoprecipitated_binned_file = "Cal27.WT.H3K36me2.10kb.bed",
    use_input = TRUE, depth_norm = TRUE, input_binned_file = "Cal27.WT_input.H3K36me2.10kb.bed",
    raw_count_cutoff = 0, pseudocount = 1e-15, scaling_factor = 0.450328805)
# generate normalized bigwig for NSD1-KO sample
norm_bw(out_dir = "output_directory", genome_assembly = "hg38",
    immunoprecipitated_binned_file = "Cal27.NSD1_KO.H3K36me2.10kb.bed",
    use_input = TRUE, depth_norm = TRUE, input_binned_file = "Cal27.NSD1_KO_input.H3K36me2.10kb.bed",
    raw_count_cutoff = 0, pseudocount = 1e-15, scaling_factor = 0.192272095)
```

# Generating scatterplots with bins stratified into genic and intergenic regions

After normalizing and converting the binned BED files into bigwigs,
we can read them in and generate a scatterplot comparing two cell lines.
The scatterplot will annotate genic and intergenic regions accordingly.
The resulting figures will be found in `/output_directory`
for the code chunk below.

Genic and intergenic regions for `hg38` and
`mm10` are included with the package and can be accessed as
indicated in the code chunk below.  **Note: you will need to adjust the
minimum and maximum values for the x- and y-axis accordingly. To find
optimal values for the x- and y-axis, run `pre_clust` to
create an exploratory scatterplot of the samples you’re
comparing.**

```
genic_intergenic_scatterplot(out_dir = "output_directory", genome_assembly = "hg38",
    cell_line = "Cal27", wildtype_samp_norm_bw = "Cal27.WT.H3K36me2.10kb.norm.bw",
    treated_samp_norm_bw = "Cal27.NSD1_KO.H3K36me2.10kb.norm.bw",
    are_R_objects = FALSE, histone_mark = "H3K36me2", output_filename = "Cal27.WT_NSD1KO.H3K36me2.10kb",
    title_of_plot = "Cal27 ChIP-seq H3K36me2", xaxis_label = "WT",
    yaxis_label = "NSD1_KO", max_x = 1, max_y = 1, min_x = -5,
    min_y = -5, pow = 1.25, show_scales = FALSE, show_legend = TRUE,
    legend_pos = "left")
```

# Exploratory analyses

## PCA

Plots of principal component analysis can be generated using
`plot_PCA` with an example below. PCA plots are useful for
assessing the treatment effect and the consistency of replicates.

```
plot_PCA(
  out_dir = "outdir",
  output_filename = "PCA_plot",
  colors = c("blue", "blue3", "red", "red4", "forestgreen", "green1"),
  sample_labels = c("samp_1", "samp_2", "samp_3", "samp_4", "samp_5", "samp_6"),
  plot_title = "",
  plot_height = 8,
  plot_width = 12,
  "samp1_10kb.norm.bw",
  "samp2_10kbb.norm.bw",
  "samp3_10kbb.norm.bw",
  "samp4_10kbb.norm.bw",
  "samp5_10kbb.norm.bw",
  "samp6_10kbb.norm.bw",
)
```

## Correlation plots

Correlation plots can be generated using
`plot_correlation` with an example below. Correlation plots
are useful for assessing separation of samples according to treatment
and consistency of replicates.

```
plot_correlation(
  out_dir = "outdir",
  output_filename = "correlation_plot",
  sample_labels = c("samp_1", "samp_2", "samp_3", "samp_4", "samp_5", "samp_6"),
  colors = c("blue", "blue3", "red", "red4", "forestgreen", "green1"),
  plot_height = 4,
  plot_width = 4,
  "samp1_10kb.norm.bw",
  "samp2_10kbb.norm.bw",
  "samp3_10kbb.norm.bw",
  "samp4_10kbb.norm.bw",
  "samp5_10kbb.norm.bw",
  "samp6_10kbb.norm.bw"
)
```

# Combining replicates

Replicates for a given treatment can be combined using
`merge_norm_bw`. The merged bigWig file can be used for
downstream analysis.

```
merge_norm_bw(
  rep1 = "WT_rep1_10kb_norm.bw",
  rep2 = "WT_rep2_10kb_norm.bw",
  merged_samples_label = "merged_WT",
  out_dir = "outdir"
)
```

# Identifying clusters of similarly-behaving genomic bins

## Processing bigwig files for clustering

Prior to generating clusters, we need to pre-process the normalized
bigWig files, which entails finding overlapping regions between two
bigWig files, removing the bottom and top 1% of bins across the two
samples (trimming to make the graphics more visible) and finally
generating a matrix of scores as well as a BED file of coordinates to be
used with HDBScan for generating clusters of similarly-behaving bins.
This is accomplished using the `pre_clus()` function. We will
continue to use the samples from the previous steps above.

**Important note: Normally, bigWig files are used as input
throughout the workflow. However, R objects of the bigWig files can also
be used, but must specified by setting
`are_R_objects=TRUE`**.

```
pre_clust(
  out_dir = "output_directory",
  treated_samp_norm_bw = "Cal27.WT.H3K36me2.10kb.norm.bw",
  wildtype_samp_norm_bw = "Cal27.NSD1_KO.H3K36me2.10kb.norm.bw",
  output_filename = "Cal27.WT.NSD1_KO.H3K36me2.10kb",
  are_R_objects = FALSE
)
```

## Matrix and pooled BED file of genomic coordinates

The resulting `mat.csv` and `pooled.bed` from
the `pre_clust()` function can be used to retrieve a matrix
of normalized signals for the two samples being compared at a given
genomic window, as shown below.

```
genomic_coordinates <- data.table::fread("pooled.bed")
signals <- data.table::fread("mat.csv") %>% `names<-`(c("KO", "WT"))
normalized_signal_matrix <- cbind(genomic_coordinates, signals)
```

## Running HDBSCAN to generate clusters

The `clus()` function can be used to generate clusters of
similarly-behaving genomic bins. It is based on an unsupervised learning
algorithm to find clusters, or dense regions, of a dataset. More details
on the Hierarchical Density-Based Spatial Clustering of Applications
with Noise (HDBSCAN) algorithm can be found
here.
The function uses `reticulate` as an interface to a Python
session. **Python version 3.9 will need to be installed in your
environment prior to running the function.** No other actions are
required from the user, as the minimal Python packages are automatically
installed in an isolated virtual environment specifically designated for
the ChIPbinner R package. This minimizes the risk of inadvertently
disrupting another Python installation on the user’s system.

**It is important to consider this step will require substantial
processing memory as well as time** . Thus, it is recommended that you
use the python-implementation of HDBSCAN in a High-performance Computing
server instead. Nevertheless, an example workflow for running HDBSCAN is
found below. Here, we use the complete matrix file, which required
substantial processing memory and multiple parallel cores, typically
found in a high-performance computing cluster. The complete matrix file
can be found at the
ChIPbinner
database.

```
# using the complete matrix
generate_clust(
  output_file_name = "Cal27.WT.NSD1_KO.H3K36me2.10kb",
  out_dir = "output_directory",
  matrix_file = "Cal27.WT.NSD1_KO.H3K36me2.10kb.mat.csv",
  minpts = 5000,
  minsamps = 5000,
  cores = 6
)
```

## Annotating clusters

After identifying clusters using HDBSCAN, these clusters need to be
annotated for use in downstream analysis. The resulting R object
contains genomic coordinates for each cluster, which can be accessed by
referencing the elements within the object list, as demonstrated below.
Each cluster will be in a
GRanges
format.

```
annotate_clust(
  number_of_clusters = 3,
  matrix_file = "Cal27.WT.NSD1_KO.H3K36me2.10kb_mat.csv",
  pooled_bed_file = "Cal27.WT.NSD1_KO.H3K36me2.10kb_pooled.bed",
  hdbscan_output_file = "clus.Cal27.WT.NSD1_KO.H3K36me2.10kb.5000.5000.txt",
  output_filename = "Cal27.WT.NSD1_KO.H3K36me2.10kb",
  out_dir = "output_directory"
)
# access each cluster, which will be in the GRanges format
cluster_A <- cons$A
cluster_B <- cons$B
cluster_C <- cons$C
```

## Generating density-based scatterplots

Finally, a density-based cluster scatterplot (along with the
genic/intergenic scatterplot) can be generated using
`density_based_scatterplot()`. Here, we used the complete
matrix of enrichment scores to generate the HBDSCAN output. The complete
HDBSCAN output can found at
ChIPbinner
database.The user can choose to include an additional density plot
that displays only the number of bins in non-annotated regions with the
parameter  `include_additional_density_plot = TRUE` .
Otherwise, this defaults to `FALSE`.

```
density_based_scatterplot(
  out_dir = "output_directory",
  genome_assembly = "hg38",
  are_R_objects = FALSE,
  output_filename = "Cal27.WT.NSD1_KO.H3K36me2.10kb",
  wildtype_samp_norm_bw = "Cal27_WT_H3K36me2_10kb.norm.bw",
  treated_samp_norm_bw = "Cal27_NSD1_KO_H3K36me2_10kb.norm.bw",
  cell_line = "Cal27",
  histone_mark = "H3K36me2",
  annotated_clusters = "Cal27.WT.NSD1_KO.H3K36me2.10kb_annotated_clusters.rda",
  number_of_clusters = 3,
  title_of_plot = "H3K36me2",
  pow = 1.1,
  min_x = -5,
  min_y = -5,
  max_x = 2,
  max_y = 2,
  hexbins = 50,
  show_scales = FALSE,
  xaxis_label = "WT",
  yaxis_label = "NSD1_KO",
  height_of_figure = 6,
  width_of_figure = 15,
  include_additional_density_plot = TRUE,
  filter_extreme_bins = TRUE
)
```

# Differential binding analysis

If replicates are included per group, differential binding (DB) can
be performed by the user. ChIPbinner uses the
ROTS
method to assess DB between two groups for each bin. Unlike methods
relying on a fixed predefined statistical model, ROTS optimizes the test
statistic directly from the data. This adaptive approach, based on
t-type statistics, ranks genomic features based on the strength of
evidence for differential binding in two-group comparisons. A priori
assumptions about the data distribution or specific cutoffs for ranking
do not need to be specified for ROTS. The optimization process maximizes
the overlap of top-ranked features in bootstrap datasets that maintain
the original group structure.

Previous performance comparisons demonstrated that ROTS, while
exhibiting less power in datasets with a small proportion of
differentially expressed features, surpasses other differential
expression methods, such as edgeR and DESeq2, in datasets characterized
by a large proportion of differentially expressed features and a skewed
distribution of these features – conditions frequently observed in
ChIP-seq data following mutations affecting global histone levels.

**Running `differentialBinAnalysis` requires significant
computational resources, including dedicated compute clusters with ample
memory** . The `bootstrap_value` parameter specifies the
number of permutation resamplings to estimate the null distribution of
the test statistic (default 1000). Increasing this value can improve the
precision of your results, but at the expense of greater computational
run-time.The `K_value` parameter specifies the largest top
list size considered. It is recommended that the K value should be
considerably higher than the number of features expected to be
differentially affected. However, increasing this value will also
substantially increase the computational run-time.

```
differentialBinAnalysis(out_dir, genome_assembly = "hg38", treated_sample_bigWigFiles = c("Cal27_NSD1KO_H3K36me2_rep1.10kb_norm.bw",
    "Cal27_NSD1KO_H3K36me2_rep2.10kb_norm.bw"), wildtype_sample_bigWigFiles = c("Cal27_WT_H3K36me2_rep1.10kb_norm.bw",
    "Cal27_WT_H3K36me2_rep2.10kb_norm.bw"), treated_condition_label = "NSD1KO",
    wildtype_condition_label = "WT", bootstrap_value = 1000,
    K_value = 1e+05, annotated_clusters = "annotated_clusters.rda")
```

# Overlap enrichment/depletion analysis with annotated clusters of bins

After identifying and annotating clusters of bins, a Fisher’s exact
test can be used to determine whether these bins overlap a specific
class of annotated regions against a background of all bins. The
underlying algorithm is based on
LOLA
- locus overlap analysis for enrichment of genomic ranges. The
example below evaluates overlap with
Ensembl
annotations genome-wide. However, the user can also evaluate overlap
with other functional annotations by inputting an R object of the
database of their choice in the parameter
`functional_db`.

The
ChIPbinner
database contains curated databases ready for input into the
parameter `functional_db` within the
`enrich_clust()` function.

Nevertheless, by specifying the `region` to be either
`genic` or `intergenic`, the user can evaluate
exclusively genic or intergenic bins overlapping a specific class of
annotated regions. In these cases, the background is stratified to only
genic or intergenic regions to avoid spurious associations to
annotations confounded by their predominantly genic or intergenic
localization.

Below is an example of how to calculate overlap enrichment &
depletion result of Ensembl annotations with bins found in cluster B
identified from the previous analyses. Note that the size of the dots
corresponds to the number of bins overlapping the corresponding
annotation. The p-value is based on Fisher’s exact test of bins
overlapping a specific class of annotated regions versus a background of
all bins found in both cell lines being compared.

```
enrich_clust(
  genome_assembly = "hg38",
  annotated_clusters = "Cal27.WT.NSD1_KO.H3K36me2.10kb_annotated_clusters.rda",
  query_cluster = "B",
  pooled_bed_file = "Cal27.WT.NSD1_KO.H3K36me2.10kb_pooled.bed",
  functional_db = "hg38_ensemblDB.rda",
  region = "genome_wide",
  cores = 1,
  n_elements = 7,
  cutoff_for_overlap = 1000,
  file_plot_name = "ensembl.genome_wide.enrichment_depletion",
  output_table_name = "ensembl.genome_wide.enrichment_depletion",
  width_of_plot = 7,
  height_of_plot = 3,
  out_dir = "output_directory"
)
```
